# Pretreatment with or without GnRH-agonist before frozen–thawed embryo transfer in patients with PCOS: a systematic review and meta-analysis

**DOI:** 10.1186/s13048-024-01410-7

**Published:** 2024-06-21

**Authors:** Jie Li, Zhong Lin, Sien Mo, Shujia Wang, Yanmei Li, Qiuling Shi

**Affiliations:** 1 State Key Laboratory of Ultrasound in Medicine and Engineering, College of Biomedical Engineering, Chongqing Medical University, Chongqing, 400016 China; 2 The Reproductive Hospital of Guangxi Zhuang Autonomous Region, Nanning, China; 3 School of Public Health, Chongqing Medical University, Chongqing, China

**Keywords:** GnRH-agonist, PCOS, Frozen embryo transfer

## Abstract

**Purpose:**

This study was aimed to systematically evaluate the efficacy of artificial cycle-prepared frozen–thawed embryo transfer (FET) with or without gonadotrophin-releasing hormone agonist (GnRH-a) pretreatment for women with polycystic ovary syndrome (PCOS).

**Methods:**

The analysis was carried out by searching the PubMed, EMBASE, and CNKI databases with a combination of keywords before October 2021. The available studies of the effects of GnRH-a pretreatment or no pretreatment on FET in PCOS patients were considered. The risk ratios (RRs) or standardized mean differences (SMD) with 95% confidence intervals (CIs) were calculated with using subgroups and sensitivity analysis. The quality evaluation for this analysis was followed.

**Results:**

Seventeen studies including 3646 women were analyzed. GnRH-a pretreatment was significantly associated with a higher implantation rate (RR = 1.12, 95%CI: 1.00–1.24) and clinical pregnancy rate (RR = 1.19, 95%CI: 1.08–1.32) than the placebo. Moreover, in the GnRH-a pretreatment group, significant differences were detected for increasing the endometrium thickness among PCOS patients (SMD = 0.56, 95%CI: 0.20–0.92). However, for RCTs subgroup, no differences were observed, even after sensitivity analyses. In addition, the miscarriage rates, ectopic pregnancy rates, multiple pregnancy rates, and live birth rates were similar in both two groups.

**Conclusions:**

Endometrial preparation using GnRH agonist pretreatment prior to FET seems to be the better choice for PCOS patients. However, well-designed RCTs are required for confirmation.

## Introduction

The endometrial receptivity and the coordination between endometrium and embryo development are the key points for implantation [1, 2]. However, the issue of how to prepare the endometrium before frozen-thawed embryo transfer (FET) to improve pregnancy outcomes remains uncertain, especially for polycystic ovary syndrome (PCOS) women [3–5]. The conventional artificial cycles are used frequently for the purpose of preparing the endometrium for PCOS women by exogenous estrogen and progesterone administration [6–8]. However, the low fertility rate and high miscarriage rate in PCOS patients indicates poor endometrial receptivity and endometrial dysfunction, when compared with the healthy women [9]. The defect in endometrial receptivity in PCOS patients is associated with the high level of androgen, which can result in poor oocyte quality and endometrial receptivity for implantation, leading to low fertilization and high miscarriage rates [9, 10].

Gonadotrophin-releasing hormone (GnRH) agonist is synthesized in the hypothalamus, which is a decapeptide hormone transiently suppressing the hypothalamic–pituitary–gonadal axis to induce a hypo-estrogenic effect, and therefore regulating the endometrial receptivity [11]. Moreover, when using GnRH agonists, not only the levels of estrogen but also the androgen can be decreased by down-regulation of GnRH receptors in the pituitary gland [12]. In addition, it can maintain lower estrogen levels after down-regulation and the shutdown of “implantation window” in advance can be prevented. Several researches have presented that PCOS is an endocrine disease with excessive production of luteinizing hormone (LH) and a hyper-androgenic microenvironment as well as the role of inflammatory factors, and the endometrial receptivity can be affected [13, 14]. Therefore, pretreatment with GnRH-a in PCOS patients may be effective for embryo implantation by adjusting the levels of estrogen and LH. In 1991, Muasher SJ et al. [15] showed that preparing the endometrium with estrogen and progesterone replacement therapy for patients undergoing FET, the higher clinical pregnancy rate was observed in women with irregular menstrual cycles or ovulation disorders when using leuprolide acetate to suppress pituitary. A few studies [16, 17] have also demonstrated GnRH-a pretreatment for PCOS patients could improve pregnancy outcomes following FET, including increasing clinical pregnancy rate and decreasing the miscarriage rate. However, other studies [18, 19] suggested that no benefits of pretreatment with GnRH-a were on improving pregnancy outcomes for PCOS patients receiving FET, but significantly increasing costs for patients.

In order to address this controversial problem, a large number of studies focusing on the efficacy of pretreatment with GnRH-a before FET in women with PCOS were conducted in the last decade. Based on the published data, it was considered necessary to conduct a persuasive systematic review and meta-analysis by stratifying patients according to the different study design types. Thus, the purpose of our study was to explore if the pretreatment with GnRH-a before FET could improve the pregnancy outcomes in a large PCOS patient population.

## Materials and methods

### Data collection and search strategy

The PubMed, EMBASE, and China National Knowledge Infrastructure (CNKI) databases were searched exhaustively for researches that explored the efficacy of the pretreatment with GnRH-a before FET for PCOS patient, with the following keywords combined: "down regulation", "GnRH agonist", "gonadotrophin-releasing hormone agonist", "polycystic ovary syndrome", "PCOS", “FET”, and “frozen-thawed embryo transfer”. The last retrieval was carried out in October 2021 and no restriction was placed on the language.

In this systematic review and meta-analysis, randomized controlled trials (RCTs) and case–control studies that compared with and without GnRH-agonist pretreatment before FET in patients with PCOS were considered. According to different preparations for endometrium, patients treated with conventional estrogen and progesterone replacement were control group. In the case group, GnRH-agonist pretreatment were administrated combined with estrogen and progesterone replacement.

### Eligibility and exclusion criteria

The inclusion criteria were defined as follows: (i) RCTs or case–control studies focused on the effects of pretreatment with or without GnRH-agonist before FET; (ii) studies on infertile patients with PCOS, and patients were diagnosed with PCOS according to two of the three Rotterdam 2003 criteria: oligoovulation or anovulation, clinical and/or biochemical signs of hyperandrogenism, polycystic ovaries [20]. For Chinese population, menstrual abnormalities combined with either hyperandrogenism or polycystic ovaries were used to PCOS diagnosis according to modified Rotterdam criteria. (iii) studies assessing at least one of the following outcomes: endometrial thickness on first day of progesterone supplementation, implantation rates, clinical pregnancy rates, miscarriage rates, ectopic pregnancy rates, multiple pregnancy rates, and live birth rates.

The major exclusion criteria were as follows: (i) studies that were not RCTs or case–control trials; (ii) studies evaluating only other clinical outcomes and not including a control group; (iii) raw data and not accessible. In addition, case reports, review articles, commentaries, and letters were all also excluded.

### Quality assessment

The quality of the included RCT studies was assessed according to the recommended approach of the Cochrane risk-of-bias tool [21]. Six specific domains were summarized: adequate sequence generation, allocation concealment, blinding, incomplete outcome data addressed, free of selective reporting, and other issues. On the premise that the results of each quality evaluation item are "yes", the quality evaluation grade of this study is considered as A. If the result of at least one quality evaluation item is "unclear" and none is "no", it is B. In addition, the quality of the case–control studies was also assessed systematically by using the criteria identified.

### Clinical outcomes and subgroup analysis

In this systematic review, the primary outcomes were endometrial thickness on first day of progesterone supplementation, implantation rates, clinical pregnancy rates, and miscarriage rates per FET cycle. In addition, ectopic pregnancy rates, multiple pregnancy rates, and live birth rates were also assessed in detail. Few studies reported on chemical pregnancy rates or endometrial thickness on the day of embryo transfer. Furthermore, the subgroups of implantation rates, clinical pregnancy rates, and miscarriage rates were analyzed according to the different types of study design, including the RCT group and case–control group.

### Statistical analysis

Using the fixed-effects and random-effects models, the pooled risk ratios (RRs) and the standardized mean difference (SMD) with 95% confidence intervals (CIs) were calculated for clinical outcomes. Forest plots were used graphically when the pooled RR estimates on the effect of pretreatment with or without GnRH-agonist were chosen before FET. In addition, the Cochrane’s Q and *I*^2^ statistic were applied to estimate heterogeneity and *P* < 0.05 was considered statistically significant [22]. Fixed-effects model was applied, when values for* I*^2^ less than 50% indicate low or moderate heterogeneity. If not, the random-effects model was applied under conditions of high heterogeneity (*I*^2^ > 50%). According to the different types of study design, subgroup analyses were carried out to further explore the source of heterogeneity. When heterogeneity was present, a sensitivity analysis was carried out by removing the study with the highest potential heterogeneity.

All of the analyses in this study were conducted with Stata version 9.0 (Stata Corporation, USA). Begg’s unweighted regression test and funnel plots were used to test for potential publication bias graphically by measuring asymmetry and drawing a vertical line (*P* > 0.05).

## Results

### Study identification and quality assessment

**Fig. 1:**
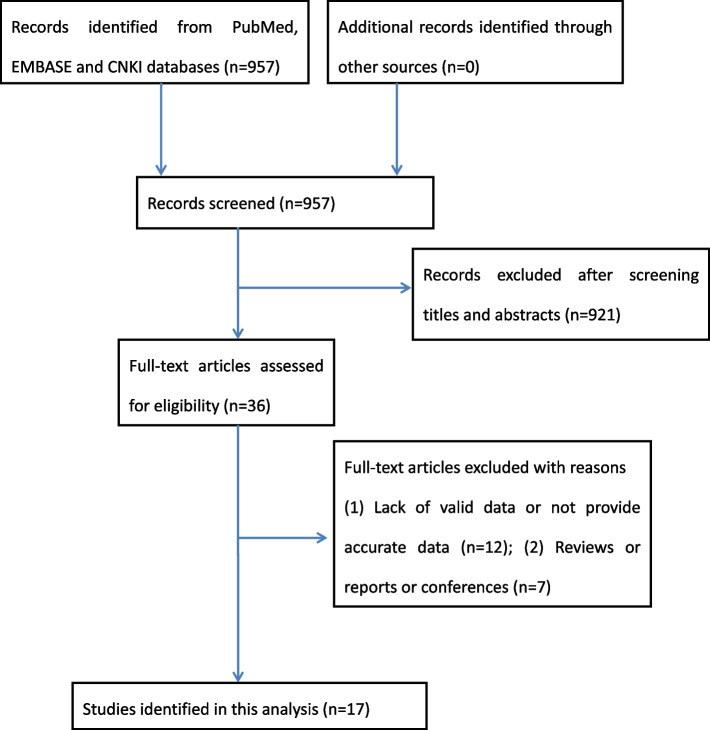
Articles identification for the process flow diagram

A total of 957 records were screened from the PubMed, EMBASE, and CNKI databases. After screening of the titles and abstracts, 921 records were excluded for not meeting the criteria and 36 studies remained for detailed full-text evaluation. 19 articles were excluded for the following reasons: 12 studies lacked valid data or did not compare pretreatment with or without GnRH-agonist before FET and 4 were reviews, reports, or conferences. Finally, 17 studies [16–19, 23–35] containing 3646 participants were eligible. The process flow diagram of selected studies is presented in Fig. 1. Comparing the effectiveness of pretreatment with or without GnRH-agonist for PCOS on clinical outcomes before FET, four RCTs [16, 18, 19, 29] and thirteen case–control studies [17, 23–28, 30–35] were analyzed. In the control group, simple artificial cycle regimen was applied for PCOS patients who received standard treatment for endometrial preparation using estradiol valerate before embryo transfer. The pretreatment group was treated with GnRH-a down-regulated artificial cycle regimen, patients with PCOS received a depot of long-acting GnRH agonist before beginning exogenous hormone supplementation. The characteristics of patients included in the analysis are summarized in Table 1.

**Table 1:** Characteristics of included studies

Authors	year	Study design	Patients /cycles		Interventions		Basic information
			Cases	Controls	Cases	Controls	
Shabnam Salemi et al	2021	RCT	93	95	Endometrial preparation with GnRH-a pretreatment	Endometrial preparation without GnRH-a pretreatment	Women with PCOS undergoing FET cycles; Age: < 37 years; BMI: unknown; Normal uterine cavity
Zhang Jianmei et al	2020	Case-controlled	79	67	Endometrial preparation with GnRH-a pretreatment	Endometrial preparation without GnRH-a pretreatment	Women with PCOS undergoing FET cycles; Age: from 21 to 35 years; BMI: between 18 and 29 kg/m^2^; Normal uterine cavity
Aghahoseini Marieh et al	2020	RCT	88	90	Endometrial preparation with GnRH-a pretreatment	Endometrial preparation without GnRH-a pretreatment	Women with PCOS undergoing FET cycles; Age: from 18 to 40 years; BMI: ≤ 30 kg/m^2^; Normal uterine cavity
L Luo et al	2020	RCT	172	171	Endometrial preparation with GnRH-a pretreatment	Endometrial preparation without GnRH-a pretreatment	Women with PCOS undergoing FET cycles; Age: from 20 to 40 years; BMI: unknown; Normal uterine cavity
Zhang Fan	2020	Case-controlled	65	72	Endometrial preparation with GnRH-a pretreatment	Endometrial preparation without GnRH-a pretreatment	Women with PCOS undergoing FET cycles; Age: < 38 years; BMI: unknown; Normal uterine cavity
Bai Jingying et al	2020	Case-controlled	124	369	Endometrial preparation with GnRH-a pretreatment	Endometrial preparation without GnRH-a pretreatment	Women with PCOS undergoing FET cycles; Age: from 20 to 34 years; BMI: unknown; Normal uterine cavity
Zhu Aizhen et al	2020	Case-controlled	32	38	Endometrial preparation with GnRH-a pretreatment	Endometrial preparation without GnRH-a pretreatment	Women with PCOS undergoing FET cycles; Age: unknown; BMI: unknown; Normal uterine cavity
Sun Xiaoxiao et al	2019	Case-controlled	221	332	Endometrial preparation with GnRH-a pretreatment	Endometrial preparation without GnRH-a pretreatment	Women with PCOS undergoing FET cycles; Age: unknown; BMI: unknown; Normal uterine cavity
Li Jing et al	2019	Case-controlled	65	65	Endometrial preparation with GnRH-a pretreatment	Endometrial preparation without GnRH-a pretreatment	Women with PCOS undergoing FET cycles; Age: from 23 to 38 years; BMI: between 22 and 27 kg/m^2^; Normal uterine cavity
He Xiao et al	2019	RCT	18	14	Endometrial preparation with GnRH-a pretreatment	Endometrial preparation without GnRH-a pretreatment	Women with PCOS undergoing FET cycles; Age: < 35 years; BMI: unknown; Normal uterine cavity
Ji Xiaoyuan et al	2019	Case-controlled	225	347	Endometrial preparation with GnRH-a pretreatment	Endometrial preparation without GnRH-a pretreatment	Women with PCOS undergoing FET cycles; Age: ≤ 39 years; BMI: unknown; Normal uterine cavity
Di XIE et al	2018	Case-controlled	48	98	Endometrial preparation with GnRH-a pretreatment	Endometrial preparation without GnRH-a pretreatment	Women with PCOS undergoing FET cycles; Age: unknown; BMI: unknown; Normal uterine cavity
Hsiao-Wen Tsai et al	2017	Case-controlled	29	31	Endometrial preparation with GnRH-a pretreatment	Endometrial preparation without GnRH-a pretreatment	Women with PCOS undergoing FET cycles; Age: from 20 to 45 years; BMI: unknown; Normal uterine cavity
Zhang Jingshun et al	2017	Case-controlled	76	76	Endometrial preparation with GnRH-a pretreatment	Endometrial preparation without GnRH-a pretreatment	Women with PCOS undergoing FET cycles; Age: unknown; BMI: unknown; Normal uterine cavity
Wang Xiaoyan et al	2015	Case-controlled	53	58	Endometrial preparation with GnRH-a pretreatment	Endometrial preparation without GnRH-a pretreatment	Women with PCOS undergoing FET cycles; Age: unknown; BMI: unknown; Normal uterine cavity
Jiang Chenglong et al	2015	Case-controlled	92	98	Endometrial preparation with GnRH-a pretreatment	Endometrial preparation without GnRH-a pretreatment	Women with PCOS undergoing FET cycles; Age: unknown; BMI: unknown; Normal uterine cavity
Jie Di et al	2014	Case-controlled	51	88	Endometrial preparation with GnRH-a pretreatment	Endometrial preparation without GnRH-a pretreatment	Women with PCOS undergoing FET cycles; Age: ≤ 35 years; BMI: ≤ 30kg/m^2^; Normal uterine cavity

*RCT* Randomized controlled trial, *BMI* Body mass index, *PCOS* Polycystic ovary syndrome, *FET* Frozen–thawed embryo transfer, *GnRH-a* Gonadotropin-releasing hormone agonist

Ultimately, quality evaluation of articles was carried out including four RCTs and thirteen case–control studies. The quality of one RCT study was for level A, other three studies for level B. The quality assessment for RCT studies was summarized in Tables 2 and 3 for case–control studies.

**Table 2:** Quality assessment for randomized controlled studies

Study	year	Study design	Adequate sequence generation	Allocation concealment	Blinding	Incomplete outcome data addressed	Free of selective reporting	Other issues	Quality assessment
Shabnam Salemi et al	2021	RCT	Yes	Yes	Unclear	Yes	Yes	Yes	B
Aghahoseini Marieh et al	2020	RCT	Yes	Yes	Yes	Yes	Yes	Yes	A
L Luo et al	2020	RCT	Yes	Yes	Unclear	Yes	Yes	Yes	B
He Xiao et al	2019	RCT	Yes	Yes	Unclear	Yes	Yes	Yes	B

A: The result of every quality evaluation item is "yes". B: At least one result of the quality evaluation item is "unclear" and none is "no"

**Table 3:** Quality assessment for case-controlled studies

Study	year	Study design	Quality assessment
Zhang Jianmei et al	2020	Case-controlled	1:adequate;2:adequate;3:adequate;4:adequate;5:adequate
Zhang Fan	2020	Case-controlled	1:adequate;2:adequate;3:adequate;4:adequate;5:adequate
Bai Jingying et al	2020	Case-controlled	1:adequate;2:adequate;3:adequate;4:adequate;5:adequate
Zhu Aizhen et al	2020	Case-controlled	1:adequate;2:adequate;3:adequate;4:adequate;5:adequate
Sun Xiaoxiao et al	2019	Case-controlled	1:adequate;2:adequate;3:adequate;4:adequate;5:adequate
Li Jing et al	2019	Case-controlled	1:adequate;2:adequate;3:adequate;4:adequate;5:adequate
Ji Xiaoyuan et al	2019	Case-controlled	1:adequate;2:adequate;3:adequate;4:adequate;5:adequate
Di XIE et al	2018	Case-controlled	1:adequate;2:adequate;3:adequate;4:adequate;5:adequate
Hsiao-Wen Tsai et al	2017	Case-controlled	1:adequate;2:adequate;3:adequate;4:adequate;5:adequate
Zhang Jingshun et al	2017	Case-controlled	1:adequate;2:adequate;3:adequate;4:adequate;5:adequate
Wang Xiaoyan et al	2015	Case-controlled	1:adequate;2:adequate;3:adequate;4:adequate;5:adequate
Jiang Chenglong et al	2015	Case-controlled	1:adequate;2:adequate;3:adequate;4:adequate;5:adequate
Jie Di et al	2014	Case-controlled	1:adequate;2:adequate;3:adequate;4:adequate;5:adequate

Quality assessment codes: 1 = including laboratory design and methods; 2 = definition of PCOS; 3 = assessment and validation of cases and controls; 4 = eliminating confounding factors for participants; 5 = Equal assessment for confounding factors for cases and controls

### Endometrial thickness

**Fig. 2:**
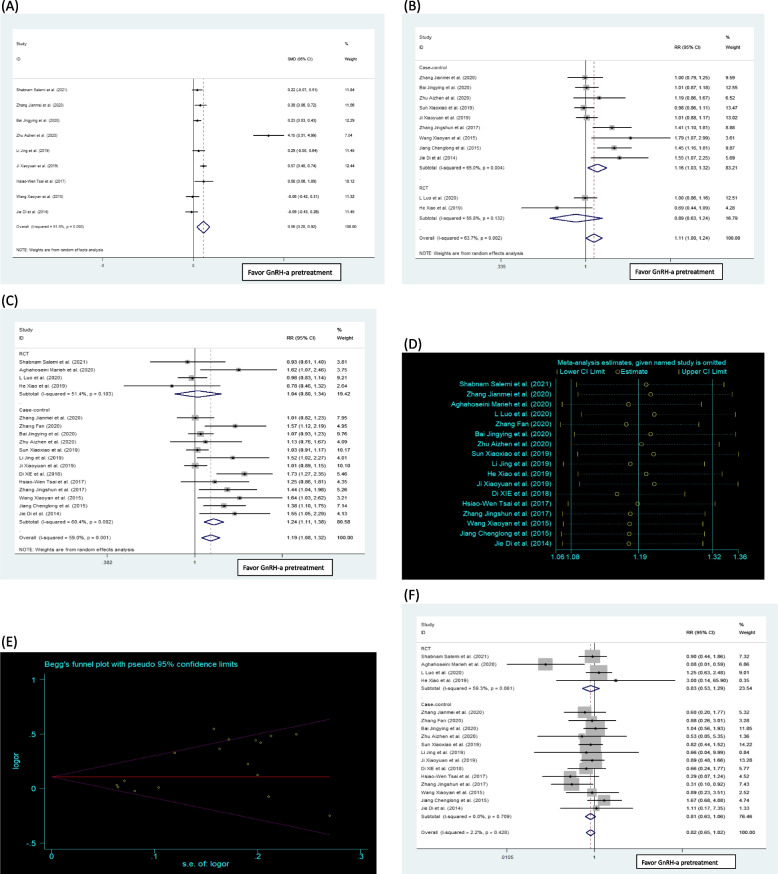
The pooled RRs or the SMD with 95% CIs of the relationship between FET cycles with or without GnRH agonist pretreatment for endometrial thickness on first day of progesterone supplementation (**A**), implantation rates (**B**), clinical pregnancy rates with random models (**C**), sensitivity analysis (**D**) and funnel plots for clinical pregnancy rate (**E**), and miscarriage rates with fixed model (**F**)

As for the effect of GnRH-a pretreatment before FET for PCOS patients, nine studies [18, 23, 25, 26, 28, 30, 31, 33, 35] with 1915 participants were included to evaluate endometrial thickness on first day of progesterone supplementation including one RCT [18] and eight case–control studies [23, 25, 26, 28, 30, 31, 33, 35]. The pretreatment of GnRH-agonist before frozen-thawed embryo transfer was effective in increasing the endometrium thickness among infertile women with PCOS. The SMD between patients using GnRH-a pretreatment and controls was 0.56 (95% CI: 0.20, 0.92, *p* = 0.000). Significant differences were detected in the GnRH-a pretreatment group when compared with the control group for endometrium thickness among PCOS patients (Table 4; Fig. 2A).

**Table 4:** Systematic review and meta-analysis results for included studies following FET with or without GnRH-a pretreatment

		Fixed/Random model		
Outcomes	Numbers of participants	SMD/RR(95% CI)	*I*^2^ (%)	Heterogeneity (*P*)
Endometrial thickness on first day of progesterone supplementation (mm)	1915	0.56(0.20,0.92)	91.9	0.000
Implantation rates	2807	1.12(1.00, 1.24)	63.7	0.002
Clinical pregnancy rates	3640	1.19(1.08, 1.32)	59.0	0.001
Miscarriage rates	2091	0.82(0.65, 1.02)	2.2	0.428
Ectopic pregnancy rates	1619	1.11(0.69, 1.78)	0.0	0.783
Multiple pregnancy rates	774	1.09(0.89, 1.33)	0.0	0.466
Live birth rates	709	1.06(0.71, 1.56)	74.0	0.009

*RR* Pooled relative risk, *SMD* Mean difference

### Implantation rate

Eleven studies [19, 23, 25–27, 29, 30, 32–35] were included in the comparison of implantation rates with a total of 2897 events with 5362 embryos transferred, and two RCTs [19, 29] and nine case–control studies [23, 25–27, 30, 32–35]. The implantation rate was 46.96% (1029/2191) in those with receiving GnRH-a pretreatment compared to 43.27% (1372/3171) in those without receiving GnRH-a pretreatment. Significant differences were detected between GnRH-a pretreatment patients and placebo patients with using the random effects model; the RR was 1.12 (95% CI 1.00–1.24, *I*^2^ = 63.7%). In the study-design subgroup analysis, a higher implantation rate was also observed in GnRH-a pretreatment group compared with the controls for the case–control study group (RR = 1.16; 95% CI: 1.03, 1.32,* I*^2^ = 65.0%). However, no significant difference was observed for the RCT group (RR = 0.89; 95% CI: 0.63, 1.24, *I*^2^ = 55.8%). To explore the high heterogeneity among studies, a sensitivity analysis was conducted. After analysis, the study with the highest heterogeneity was shown [27], which was the study only focused on PCOS population with first frozen-thawed embryo transfer transplant failure. After removing the highest heterogeneity study, the statistical difference was also similar to previous results. No significant bias was detected and the funnel plot was estimated to be symmetric using Begg’s test. (Table 5; Fig. 2B).

**Table 5:** The subgroup analysis for the primary pregnant outcomes according to study design

			Fixed/Random model			Sensitivity analysis		
Outcome	Numbers of participants	Subgroup analysis	RR(95%CI)	*I*^2^ (%)	Heterogeneity (*P*)	RR(95% CI)	*I*^2^ (%)	Heterogeneity (*P*)
Implantation	2807	RCT	0.89(0.63, 1.24)	55.8	0.132	0.89(0.63,1.24)	55.8	0.132
		Case-controlled	1.16(1.03, 1.32)	65.0	0.004	1.21(1.05,1.39)	63.6	0.007
		Total	1.12(1.00, 1.24)	63.7	0.002	1.14(1.01,1.29)	64.5	0.003
Clinical pregnancy	3640	RCT	1.04(0.81,1.34)	51.4	0.103	1.04(0.81,1.34)	51.4	0.103
		Case-controlled	1.24(1.11,1.38)	60.4	0.002	1.28(1.13,1.44)	57.9	0.006
		Total	1.19(1.08, 1.32)	59.0	0.001	1.22(1.10,1.36)	58.5	0.002
Miscarriage	2091	RCT	0.83(0.53,1.29)	59.3	0.061	0.79(0.32,1.95)	57.4	0.071
		Case-controlled	0.81(0.63,1.06)	0.0	0.709	0.87(0.66,1.15)	0.0	0.896
		Total	0.82(0.65, 1.02)	2.2	0.428	0.89(0.70,1.31)	0.0	0.619

*RR* Pooled relative risk, *RCT* Randomized controlled trial

### Clinical pregnancy rate

Seventeen studies [16–19, 23–35] involving 3640 participants were analyzed for clinical pregnancy rate including four RCTs [16, 18, 19, 29] and thirteen case–control studies [17, 23–28, 30–35]. Successful clinical pregnancy occurred in 934 of 1531 (61.01%) patients receiving GnRH-a pretreatment and in 1157of 2109 (54.86%) participants for patients without receiving pretreatment. With using the random-effects model, the results presented that the difference in clinical pregnancy rates between the GnRH-a pretreatment and no pretreatment groups was statistically significant, and the RR was 1.19 (95% CI: 1.08, 1.32, *I*^2^ = 59.0%) (Table 4; Fig. 2C). Due to the high heterogeneity for the clinical pregnancy rate, we carried out a subgroup analysis and a sensitivity analysis to explore the sources of heterogeneity. In the subgroup analysis for case–control study, a higher clinical pregnancy rate was observed in PCOS patients receiving GnRH-a pretreatment compared with the control group not receiving GnRH-a pretreatment (RR = 1.24, 95% CI: 1.11, 1.38, *I*^2^ = 60.4%). However, for the RCT group, there was no significant difference (RR = 1.04, 95% CI: 0.81, 1.34, *I*^2^ = 51.4%) (Table 5; Fig. 2C). In the sensitivity analysis, the study focusing on the GnRH-a pretreatment for PCOS population had the highest heterogeneity [30]. After removing the highest heterogeneity study, the statistical difference was also similar to previous results and no significant difference was observed for RCT group (Table 5; Fig. 2D). No significant bias was detected with using Begg’s test (Fig. 2E).

### Miscarriage rate

Seventeen studies [16–19, 23–35] reported the miscarriage rate in 2091 participants including four RCTs [16, 18, 19, 29] and thirteen case–control studies [17, 23–28, 30–35]. Miscarriage occurred in 109 of 934 (11.67%) events in the GnRH-a pretreatment group and in 162 of 1157 (14.00%) events in the control group. When the fixed-effects model was used, the pooled analysis showed no significant difference in the miscarriage rate between the two groups (RR = 0.82, 95% CI: 0.65, 1.02, *I*^2^ = 2.2%) (Table 4; Fig. 2F). In addition, the study-design subgroup analysis and sensitivity analysis showed no significant differences (Table 5). However, the heterogeneity for the RCT group was high (*I*^2^ = 59.3%) compared with the case–control study group (*I*^2^ = 0.0%). No publication bias was detected.

### Ectopic pregnancy rate

Eleven case–control studies [17, 23, 25–28, 30, 32–35] were included in the comparison of ectopic pregnancy rate for a total of 1619 patients. Ectopic pregnancy rate occurred in 21 of 686 (3.06%) events in the pretreatment of GnRH-a patients and in 39 of 933 (4.18%) events in the no-pretreatment patients. No significant differences were found between the case and control groups, and the RR was 1.11 (95% CI 0.69–1.78, *I* ^2^ = 0.0%) in the fixed effects model (Table 4). Using Begg’s test, we did not detect any significant bias.

### Multiple pregnancy rate

Six studies [17, 18, 23, 30, 34, 35] were included to evaluate the effect of pretreatment of GnRH-a before FET on multiple pregnancy rates with 774 participants, one RCT [18] and five case–control studies [17, 23, 30, 34, 35]. For the patients with PCOS, the multiple pregnancy rate was 34.08% (121/355) in the pretreatment of GnRH-a group, which was higher than the control group (29.83%, 125/419). However, no significant differences were found between the two groups with using the fixed effects model, and the RR was 1.09 (95% CI 0.89–1.33, *I* ^2^ = 0.0%) (Table 4). No publication bias was detected.

### Live birth rate

In the analysis, only four related studies [17–19, 29] were included in the comparison of live birth rates for a total of 709 patients including three RCTs [18, 19, 29] and one case–control study [17]. Live birth occurred in 141 of 331 (42.60%) events in the pretreatment of GnRH-a group and in 153 of 378 (40.48%) events in the no-pretreatment group. The difference between the GnRH-a pretreatment group and control group did not reach statistical significance for live birth rate, and the RR was 1.06 (95% CI 0.71–1.56, *I*^2^ = 74.0%) in the random effects model (Table 4). A sensitivity analysis was carried out considering the high heterogeneity. Similar to previous result, no significant difference was identified after removing the article with the highest heterogeneity [29]. No publication bias was detected and the funnel plot was symmetrical.

## Discussion

Comparing large samples of GnRH-a pretreatment following artificial cycle of estrogen preparing endometrium to controls for PCOS patients undergoing FET treatments, the study was aimed to explore the efficacy of GnRH-a pretreatment in PCOS patients and the function for the pregnancy outcomes. Seventeen studies with 3640 patients were included in this study. The results indicated that endometrial preparation by using GnRH agonist pretreatment before FET might be a better choice for PCOS patients. When comparing to no-pretreatment group, the endometrium thickness increased among women with PCOS after using the GnRH-agonist before. Moreover, the implantation and clinical pregnancy rates also elevated significantly for PCOS patients undergoing FET by dealing with GnRH-a. However, no significant effects were discovered for the miscarriage rates, ectopic pregnancy rates, multiple pregnancy rates, or live birth rates, even in the subgroup analyses.

PCOS is a complex endocrine disorder which is characterized by chronic anovulation and hyperandrogenism. Despite several treatments were said to improve ovulation problems, overall pregnancy rates were still not ideal. The implantation failure and spontaneous miscarriages also occurred frequently [31]. And endometrial dysfunction and hyperandrogenism might lead to the implantation failure. For the PCOS, frozen embryo transfer in a freeze-only cycle strategy was a preferred option due to the low probability of OHSS comparing to fresh transfer strategy. In order to achieve optimal synchronisation between the embryo and endometrium, a suitable endometrial preparation protocol for PCOS patients in FET cycles was important. However, little attention had been paid to the development of an appropriate endometrial preparation protocol for FET in PCOS population. Patients with PCOS are often anovulatory, simple artificial cycle regimen with using estrogens and progesterone was usually applied for PCOS patients. However, the peri-implantation embryonic and uterine development might be interfered by the high levels of testosterone resulting in implantation failure. In 2003, Cermik D et al. [36] found that *HOX10* gene was essential for endometrial development and regulated negatively by testosterone, which suggested that hyperandrogenism was related to poor endometrial receptivity in PCOS patients. Besides steroid administration, endometrial preparation for FET with GnRH-a pretreatment had also been recommended to improve implantation rate. One possible mechanism was the inhibition of endometrial inflammation and enhanced expression of endometrial adhesion molecules after using of GnRH-a pretreatment, through suppressing the serum LH, E_2_ level and GnRH–HCG axis function [16].

In 2013, a systematic review and meta-analysis including 20 studies presented inefficacy in the clinical pregnancy rate, ongoing pregnancy rate or live birth rate after using different protocols in preparing the endometrium whether pretreatment with GnRH agonist or not in FET patients [37]. Because of limited studies focused on whether GnRH-a pretreatment using for endometrial preparation in PCOS patients undergoing FET, little consensus was shown on the effective strategies of endometrium preparation protocols of FET for PCOS patients. However, for women with ovulatory cycles, recent findings might result in a change in clinical practice, towards a preference for natural cycle FET (NC-FET) over artificial cycle FET (AC-FET) cycles. In 2022, Roelens C et al. [38] carried out a retrospective cohort study which showed that a higher incidence of pre-eclampsia in AC-FET versus NC-FET (11.8% vs. 3.7%). In 2023, a meta-analysis [39] including 30 studies also suggested that NC-FET decreased the risk of adverse obstetric and neonatal outcomes comparing to AC-FET including lower rates of hypertensive disease during pregnancy and preeclampsia. Thus, preparing the endometrium for FET, we should not only take the basic question of effectiveness into account, but also consider its safety.

In our analysis, we aimed to explore the efficiency of GnRH agonist before endometrial preparation for FET especially for PCOS patients. Recently, only four RCTs had been carried out for endometrial preparation protocols about GnRH agonist pretreatment in PCOS populations undergoing FET treatments. In 2020, one of them [19] randomized 343 patients with PCOS undergoing FET for endometrial preparation to or not to receive GnRH-a pretreatment, the results showed that the similar outcomes were found in implantation rate, clinical pregnancy rate and miscarriage rate between cases and controls, only with increased cost for patients with GnRH-a pretreatment. There should be some factors for this RCT that cannot be ignored affecting the results. Regarding the ovarian stimulation protocols used in the fresh cycle, a high heterogeneity was presented. In addition, more than one FET cycle (range 1–2) per patient were included which did not rule out the interference of other factors. At the same time, another RCT [16] was carried out. And the results suggested that endometrial preparation using GnRH agonist improved ongoing pregnancy and decreased miscarriage rate by reducing androgen level in PCOS patients and improving the receptivity. The conclusion of this study [16] was supported by several retrospective studies [17, 24]. But more scholars [31–33, 35] had argued that pretreatment with GnRH agonist might improve pregnancy rates, but not miscarriage rates in PCOS women.

By including a relatively large sample size, our study was the first meta-analysis to compare artificial cycle with or without GnRH agonist pretreatment for patients with PCOS. Our data also showed that GnRH agonist pretreatment before FET had a crucial role in pregnancy outcomes for PCOS patients by increasing endometrium thickness and improving implantation rates. The strengths of our study not only included the RCTs, but also case–control studies, which could reduce the selection bias. Moreover, due to the inclusion of a large number of researches, the results of our study were more comprehensive and more reliable than other single studies. In the midst of heated debate, our findings provided a tentative answer that clinicians could use to better guide the treatment scheduling.

Although some high-quality studies with large sample sizes were included, several limitations were clear. In general, randomized case–control studies are more convincing than case–control studies. However, no significant difference had been identified in the RCTs subgroup in this analysis. Only in the case-controlled studies group, GnRH agonist pretreatment was identified to be associated with higher implantation and pregnancy rates compared to no-pretreatment patients. Nevertheless, the high heterogeneity was detected in the RCTs subgroup for miscarriage rate. Some bias might be induced with the high heterogeneity and the exact functions could not be concluded just based on this meta-analysis. Another limitation is the live birth rate, which was the end result of our follow-up. But only four studies focused on the analysis with and without GnRH agonist pretreatment. The limitation of a lack of data should require more studies to follow up live birth rates. In addition, pregnancy-related complications and neonatal outcomes were not analyzed due to the limitation of lack of data. Therefore, further researches are still required to compare the maternal and neonatal safety with using the GnRH agonist pretreatment protocol. Lastly, some confounding factors should also be taken into account in this analysis, such as the different dose of GnRH-a (1.0 mg or 1.875 mg or 3.75 mg used in studies), the different duration and the period of use of GnRH-a pretreatment, the different characteristics of patients, the different types of experimental design, and the different statistical methods. Thus, well-designed RCTs and case–control studies are needed to confirm these results.

## Conclusions

For PCOS patients, an endometrial preparation using GnRH agonist pretreatment prior to artificial cycle could improve implantation rates and clinical pregnancy rates compared with the conventional artificial cycle protocol without GnRH-a pretreatment. Therefore, artificial cycle with GnRH-a pretreatment appears to be the better choice for women with PCOS. However, well-designed RCTs are required for confirmation.
